# Better Care and Less Work? Framings of Carework in U.S. Age Tech Policy Documents

**DOI:** 10.1093/geroni/igaf122.1818

**Published:** 2025-12-31

**Authors:** Anne Barrett, Brianna Soulie, Hope Mimbs

**Affiliations:** Florida State University, Tallahassee, Florida, United States; Florida State University, Tallahassee, Florida, United States; Florida State University, Tallahassee, Florida, United States

## Abstract

As technologies aimed at enhancing older adults’ quality of life continue to advance, so does the optimism surrounding their potential to improve care. This framing appears in state policy documents that address technology and aging, but relatively few studies have examined them. Our study addresses this gap by analyzing age tech policy documents published over the past decade by major institutional actors in U.S. aging policy (e.g., Office of Science and Technology Policy; Department of Health and Human Services). Our analyses revealed that age tech is framed not only as providing older adults with better care, defined as more effective, more efficient, and safer care, but also as reducing care providers’ work by making it less time-intensive and demanding. Critical analysis of these framings, however, revealed that age tech, especially digital forms centering on monitoring and surveillance, can have the opposite effect. It expands the work of care providers, paid and unpaid, by creating new tasks, like “tech support,” and reduces time spent in more emotionally rewarding aspects of carework, like providing support in face-to-face interactions. Age tech also creates new forms of “self-care work” for older adults, like collecting and tracking biometric health data. These shifts can diminish rather than improve quality of care. We interpret these intertwined illusions of better care and less work through an analysis of the political economic underpinnings of technology’s application to carework, which reveals how it contributes to the exploitation, alienation, and commodification of care workers, as well as care recipients.

